# Real Life Blood Management Practices in Thalassemia and Myelodysplastic Syndrome Patients

**DOI:** 10.1155/anem/7257391

**Published:** 2025-01-08

**Authors:** Muruvvet S. Aydin, Esra Cengiz

**Affiliations:** ^1^Department of Hematology and Bone Marrow Transplantation, Ankara Sehir Hastanesi, Ankara, Turkey; ^2^Department of Hematology, Mehmet Akif Inan Egitim Ve Arastirma Hastanesi, Sanliurfa, Turkey

## Abstract

The effect of pretransfusion hemoglobin on transfusion burden, thrombosis, and mortality in thalassemia and myelodysplastic syndrome is unclear. We aimed to study the pretransfusion hemoglobin and erythrocyte transfusion burden and investigate the effects of these variables on each other in real-life in thalassemia and myelodysplastic syndrome. Adult patients with thalassemia and myelodysplastic syndrome who received at least one erythrocyte concentrate unit outpatient at Sanliurfa Mehmet Akif Inan Training and Research Hospital during 1 year were included in the study. The data were retrospectively obtained. Ethical approval was obtained for the study. Ninety-two patients were included in the study. In thalassemia major, pretransfusion hemoglobin ≥ 9 g/dL was associated with a lower median annual number of transfused erythrocyte concentrate units (15 vs. 27) and median annual number of transfusion sessions (11 vs. 14) *p*=0.002, *p*=0.009, respectively). In myelodysplastic syndrome, a pretransfusion hemoglobin level ≥ 8 g/dL was associated with a lower median annual number of transfused erythrocyte concentrate units (6 vs. 24) (*p*=0.016). In thalassemia major with an intact spleen, pretransfusion hemoglobin ≥ 8 g/dL was associated with an increased median annual number of transfused erythrocyte concentrate units (32 vs. 27) and median annual number of transfusion sessions (18 vs. 14) (*p*=0.046, *p*=0.038, respectively). In conclusion, higher pretransfusion hemoglobin levels were related to a lower transfusion burden in thalassemia major and myelodysplastic syndrome.

## 1. Introduction

Thalassemia is one of the most common anemia treated with red cell transfusion [1] and has a particularly high prevalence in the Middle East [2]. Patients may be treated differently depending on their country and institution [3]. It is classified into transfusion-dependent thalassemia (TDT) and non-TDT (NTDT) [2, 4]. In myelodysplastic syndrome (MDS), the need for regular erythrocyte concentrate (EC) transfusion arises as a result of anemia [5, 6], and iron overload and associated decrease in organ function, transfusion reactions, and decreased quality of life (QoL) may occur [7].

Transfusion must be safe and effective, and it should be kept in mind that the transfusion recommendations applied to other patients are not suitable for thalassemia patients [1, 8]. Experts have different pretransfusion hemoglobin (Hb) threshold, target post-transfusion Hb, and transfusion frequency recommendations for thalassemia [1, 4, 9–11]. In the Thalassemia International Federation guidelines, it is suggested that transfusion should be performed every two–four weeks to keep the pretransfusion Hb above 9–10.5 g/L or > 11–12 g/dL in thalassemia with cardiac complications [8]. In a recent study by Musallam et al., pretransfusion Hb level was the best predictor of mortality [12]. In MDS, the most appropriate pretransfusion threshold is less clear, and because of the lack of sufficient evidence on this subject, experts have developed their own practice points [13]. British Society of Hematology (BSH) suggests making patient-based decisions for transfusion by considering patient symptoms [14].

Due to the lack of similar data in real life and the lack of sufficient evidence in the literature on this subject, we hypothesized that the pretransfusion Hb threshold affects the annual EC transfusion burden (in units and sessions) in thalassemia and MDS patients. Therefore, we aimed to shed light on the future transfusion guidelines.

## 2. Materials and Methods

### 2.1. Patients

**Figure 1:**
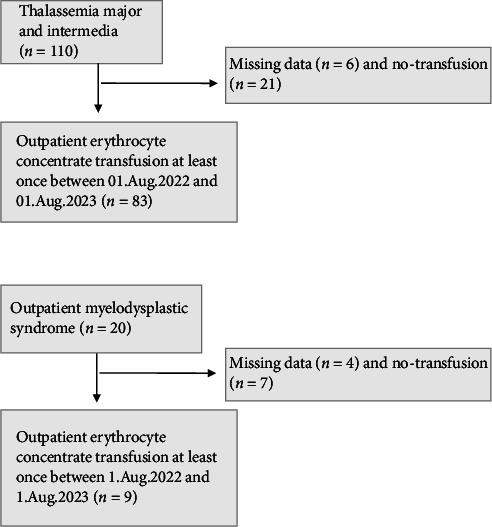
Flowchart of the patient selection.

Adult thalassemia and MDS patients who received at least one EC unit at the Sanliurfa Mehmet Akif Inan Training and Research Hospital outpatient transfusion unit during the 1-year study period were included. Patients for whom sufficient data could not be obtained were excluded from this study. As genotype determination and molecular testing are not accessible for thalassemia patients, patients with thalassemia were classified as major or intermedia according to hospital file records. MDS patients were recorded as low- or high-risk according to the revised international prognostic scoring system (R-IPSS) [15]. The patient selection process is illustrated in Figure 1.

### 2.2. Data Collection

Demographic and clinical data were obtained retrospectively from the electronic and written records of patients at Sanliurfa Mehmet Akif Inan Training and Research Hospital. To ensure data accuracy, inconsistencies were addressed and a feedback mechanism was implemented during data recording and analysis. Age, gender, comorbidities, medications used, and transfusion indications were recorded as the clinical and demographic variables. Comorbidities that may increase the need for transfusion were determined as congestive heart failure, coronary artery disease, chronic lung diseases, and chronic kidney disease. Patients with any of these diseases were classified as “those with comorbidities”. Hb level measured before transfusion, presence of anemia-related findings before transfusion, number of EC units transfused, and survival data were recorded.

### 2.3. Statistical Analysis

Statistical analyses were performed using Statistical Packages for the Social Sciences v20.0 (SPSS Inc., Chicago, IL). Descriptive analyses were performed using medians and ranges for non-normally distributed and ordinal data. In the analysis of the numerical variables, the Mann–Whitney *U* test and Kruskal–Wallis test were used because they were not normally distributed and independent. The chi-square test was used for qualitative variables of contingency tables. Spearman test was used to define correlation of non-normally distributed numerical variables. A *p* value of less than 0.05 was considered to show a statistically significant result.

### 2.4. Ethical Approval

The study was conducted ethically, and all procedures were conducted in accordance with the requirements of the World Medical Association's Declaration of Helsinki. This study was reviewed and approved by the Institutional Review Board at Harran University (HRÜ/23.19.06, 16.October.2023). Informed consent was not required because this study included retrospective observational data.

## 3. Results and Discussion

A total of 92 patients with thalassemia (major and intermedia) and MDS were transfused with EC in our center within one year. The age and gender characteristics of the patients, according to their diagnosis, are shown in Table 1.

### 3.1. Thalassemia Major

There were 67 thalassemia major patients. Medical records were insufficient for pretransfusion anemia symptoms. All patients were alive. A total of 32 patients underwent splenectomy. The median annual EC transfusion per patient was 25 (range 8–48) units, and it was statistically higher in non-splenectomized patients than in splenectomized patients (median, 29 vs. 22.5; *p*=0.007). The median annual EC transfusion session per patient was 14 (range 4–24) sessions, and it was significantly higher in non-splenectomized patients than in splenectomized patients (median 16 vs. 13, *p*=0.014). The median annual EC transfusion session did not show difference according to sex (*p*=0.095) or comorbidity (*p*=0.948), and was not correlated with age (*r* = −0.234, *p*=0.056). The median annual EC transfusion per patient did not show difference according to sex (*p*=0.061) or comorbidity (*p*=0.847), but it had a very weak negative correlation with age (*r* = −0.298, *p*=0.014) (Table 2).

The median pretransfusion Hb threshold value was 8.40 (range 5.50–9.80) and it was statistically higher in splenectomized patients than in non-splenectomized patients (median 8.6 vs. 7.8, *p* ≤ 0.001). The median pretransfusion Hb threshold did not show difference according to sex (*p*=0.436) or comorbidity (*p*=0.382), and was not correlated with age (*r* = 0.126, *p*=0.309).

When the patients were stratified according to pretransfusion Hb threshold values, the median annual EC transfusion per patient differed significantly between the groups (*p*=0.009) (Table 3). Patients with a pretransfusion Hb threshold of ≥ 9 g/dL (*n* = 5) had significantly fewer annual EC transfusions than patients with an Hb threshold < 9 g/dL (median 15 units vs. 27 units) (*p*=0.002). A significant difference was also observed in the number of annual EC transfusion sessions according to the pretransfusion Hb threshold values (*p*=0.020) (Table 3). Patients with a pretransfusion Hb threshold of ≥ 9 g/dL had significantly fewer annual EC transfusion sessions than patients with an Hb threshold of < 9 g/dL (median 11 vs. 14 sessions) (*p*=0.009). There were no thalassemia major cases with an intact spleen and pretransfusion Hb threshold of 9 g/dL. In thalassemia major with an intact spleen, pretransfusion Hb ≥ 8 g/dL was significantly associated with a higher median annual EC transfusion (median 32 vs. 27 units) (*p*=0.046) and median annual EC transfusion sessions (median 18 vs. 14 sessions) (*p*=0.038) than those with pretransfusion Hb < 8 g/dL.

### 3.2. Thalassemia Intermedia

There were 16 thalassemia intermedia patients. Medical records were insufficient about pretransfusion anemia symptoms. All patients were alive. The annual median EC transfusion per patient was 6.5 (range 4–13) units. The median annual number of EC transfusion sessions per patient was four (range 2–8) sessions. The median annual EC transfusion session and the median annual EC transfusion per patient did not show difference according to sex (*p*=0.069, *p*=0.267, respectively) and comorbidity (*p*=0.500, *p*=0.125, respectively), and was not correlated with age (*r* = 0.228, *p*=0.395; *r* = 0.365, *p*=0.165, respectively).

The pretransfusion Hb threshold was median 8.52 g/dL (range 6.80–9.40) and for pregnant patients it was median 8.70 g/dL (range 8.60–8.95). The median pretransfusion Hb threshold did not show difference according to comorbidity (*p*=0.125) and was not correlated with age (*r* = 0.141, *p*=0.603); however, it was slightly higher in females (8.6 vs. 7.2) (*p*=0.013).

When the patients were stratified according to the pretransfusion Hb threshold values, the median annual EC transfusion per patient did not differ between the groups (*p*=0.644) and this was also true for the female and male subgroups (*p*=0.258 and *p*=1.000, respectively). There was no significant difference in the number of annual EC transfusion sessions according to the pretransfusion Hb threshold (*p*=0.632) and this was also true for the female and male subgroups (*p*=0.748 and *p*=0.617, respectively) (Table 4).

Five patients were splenectomized. Annual EC transfusions per patient (median 7 vs. 6), annual EC transfusion sessions per patient (median 5 vs. 3), and the pretransfusion Hb threshold values (median 8.6 vs. 7.7) were higher in the non-splenectomized thalassemia intermedia patients than in splenectomized patients, but the differences were not statistically significant (*p*=0.432, *p*=0.202, and *p*=0.268, respectively).

### 3.3. Thalassemia Major vs Thalassemia Intermedia

Patients with thalassemia intermedia were significantly older than those with thalassemia major (*p* ≤ 0.001) (Table 1). Pretransfusion Hb threshold values were similar in thalassemia major and intermedia (median 8.40 vs. 8.52, *p*=0.840).

### 3.4. MDS

There were nine patients with MDS (three low-risk MDS and six high-risk MDS). Medical records were insufficient about pretransfusion anemia symptoms. During the 1-year period, three patients (two high-risk MDS and one low-risk MDS) died. Patients with low- and high-risk MDS were similar in terms of median age (67 vs. 73 years, *p*=0.714). The median annual EC transfusion per patient was 13 (range 5–57) units. The median annual number of EC transfusion sessions per patient was six (range 3–30) sessions.

The median annual EC consumption was similar in low- and high-risk MDS patients (*p*=0.262), did not show difference according to sex (*p*=0.413) and was not correlated with age (*r* = −0.084, *p*=0.831); however, it was significantly lower in those with comorbidities (5.5 vs. 20 units, *p*=0.032). The number of EC transfusion sessions was significantly higher in high-risk MDS patients (median 3 vs. 8.5, *p*=0.048), did not show difference according to sex (*p*=0.413) and comorbidity (*p*=0.063), and was not correlated with age (*r* = 0.008, *p*=0.983) (Table 5).

The pretransfusion Hb threshold value was median 8.20 (range 5.70–8.90). Pretransfusion Hb threshold was similar in the low- and high-risk MDS (median 8.20 vs. 7.95, *p*=0.905), was not different according to gender (*p*=0.413) and was not correlated with age (*r* = −0.167, *p*=0.667) it was found to be higher in those with comorbidities (8.4 vs. 7.2 gr/dL, *p*=0.0016). Pretransfusion Hb levels were not significantly different between the alive and deceased MDS patients (*p*=0.905).

Patients with a pretransfusion Hb threshold of ≥ 8 g/dL (*n* = 5) had significantly fewer annual EC transfusions than patients with an Hb threshold < 8 g/dL (median 6 units vs. 24 units) (*p*=0.016). Patients with a pretransfusion Hb threshold of ≥ 8 g/dL had fewer annual EC transfusion sessions than patients with an Hb threshold of < 8 g/dL (median 4 vs. 12 sessions), but the difference was not statistically significant (*p*=0.063). All patients with MDS and comorbidity had a pretransfusion Hb threshold of ≥ 8 g/dL; in patients with MDS without comorbidity, a pretransfusion Hb threshold of ≥ 8 g/dL was not related to median annual EC transfusion in units (*p*=0.400) and median annual EC transfusion sessions (*p*=0.800).

## 4. Thrombosis

There was no history of thrombosis and/or anticoagulant use in patients with thalassemia intermedia or MDS. One patient with thalassemia major had a history of pulmonary thromboembolism, with a median pretransfusion Hb of 7.6 g/dL.

**Figure 2:**
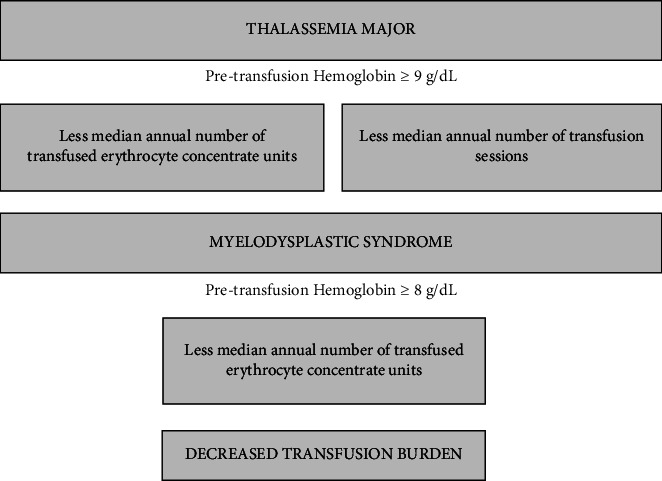
Pretransfusion Hb and transfusion burden.

The key findings of this study are shown in Figure 2. In this study, age, sex, and to have comorbidity were not major determinants of transfusion burden and pretransfusion Hb threshold in thalassemia major, a pretransfusion Hb threshold ≥ 9 g/dL was associated with a lower transfusion burden in thalassemia major. This finding is consistent with the current guidelines [11]. However, pretransfusion Hb levels in thalassemia major patients were lower than those recommended by the guidelines [16, 17]. This may be due to concerns regarding iron overload and the desire to avoid donor exposure. Although the widespread use of oral iron chelators reduces concerns about iron overload [1] there may be concerns about compliance with chelation therapy. None of our thalassemia patients was taking medications that could affect transfusion requirements, such as luspatercept, mitapivat, or hydroxyurea. However, an unknown genotypic load should be considered when interpreting these results.

Differences in transfusion practices were related to accessibility, patient compliance, doctors' attitudes, costs, and use of treatment guidelines [18]. Our patients may have problems complying with the transfusion schedule and may not come to their sessions until they feel severe fatigue. The authors state that transfusion in the same outpatient unit where patients receive chemotherapy may be disturbing, especially in thalassemia patients [2]. In our cohort, blood transfusions and chemotherapy were performed in separate units.

In thalassemia, the transfusion volume is generally recommended to be two–four units per transfusion session depending on the pretransfusion Hb level [1]. Median 1.8 and 1.9 units of EC were transfused per session in patients with thalassemia major and thalassemia intermedia, respectively. We attributed this lower transfusion volume to the fact that patients mostly applied to the transfusion center from the districts; as a result, the impossibility of transfusion of three EC units in one day and the patient's reluctance to return for a session on a different day.

Recommendations in older guidelines may not be valid for the non-splenectomized thalassemia population, and when splenectomy is no longer performed frequently, the effect of the pretransfusion Hb target on transfusion volume is not clear [1]. In the analysis of patients with a known splenectomy status, splenectomy determined the burden of EC transfusion. Non-splenectomized patients had a higher transfusion burden and lower pretransfusion Hb thresholds. In thalassemia major with an intact spleen, higher pretransfusion Hb was associated with a higher transfusion burden in our cohort.

Lower Hb level has also been associated with common complications in NTDT patients and the pretransfusion Hb levels in this study were lower than those recommended by guidelines [19]. Age, sex, and presence of comorbidity were not major determinants of transfusion burden, but higher pretransfusion Hb threshold in female thalassemia intermedia patients could be related to higher target levels in pregnant patients.

The pretransfusion Hb threshold value of 8.2 g/dL in our MDS cohort indicated that a restrictive strategy was followed. However, the pretransfusion Hb levels were higher in patients with comorbidities. If we look at the results of studies on MDS, it can be said that liberal transfusion may be preferred, especially in terms of QoL [20]. One of the important studies comparing the restrictive transfusion strategy with the liberal strategy in MDS is the study by Stanworth et al., and in this study, better QoL results were observed with the liberal strategy [21].

In a recent study, a liberal pretransfusion Hb target (11–12.5 g/dL) was associated with more transfusion visits, more blood count testing, transfusion of more blood units, and shorter time to transfusion [22]. Interestingly, a recent large study showed that patients with MDS chose to receive transfusions at lower Hb levels [23]. Experts also recommend that patient symptoms rather than pretransfusion Hb should be considered in decision-making [24]. One of the points to note is that the median age of our patient group was older. It is important to pay attention to nuances such as cardiovascular comorbidities when determining the Hb threshold values of this patient group [25].

The average annual transfusion in MDS has been reported as 7–15 EC units [25]. In our study, a median of 13 units of EC transfusions annually was within this range, which is consistent with the literature. We also observed more EC transfusion sessions in high-risk MDS patients than in low-risk MDS patients. However, we did not encounter a similar finding in the literature. The significantly lower transfusion burden in patients with MDS and comorbidities may be attributed to the higher pretransfusion Hb threshold in this cohort. Besides, it should be kept in mind that confounding variables, such as cytogenetic and molecular profile, the use of hypomethylating agents, erythropoietin-stimulating agents, or other treatments, were not evaluated in our study.

The most important limitations of this study were its retrospective nature and small number of patients with MDS. All thalassemia patients were alive, and this study was not suitable for the analysis of mortality in thalassemia. Besides, since thrombosis was reported in only 1 patient, its association with pretransfusion Hb could not be analyzed. In MDS, the pretransfusion Hb levels of deceased and alive patients were not different. Although we wanted to evaluate pretransfusion symptoms in our study, sufficient data could not be obtained. To mitigate these limitations, prospective multicenter studies should be conducted, patients' anemia symptoms should be recorded, long-term follow-up for mortality should be performed, and genetic subgroups in MDS and the effect of novel agents should be analyzed. There is a serious gap in the literature regarding cost-effectiveness, which is outside the scope of this study.

## 5. Conclusions

This study clearly showed that the current guidelines for thalassemia management may be insufficient to implement recommendations in real life and provides evidence for clinical guidelines to recommend targeting a pretransfusion Hb threshold of 9 g/dL in thalassemia major and 8 g/dL in MDS to reduce transfusion burden. In non-splenectomized thalassemia major, targeting higher pretransfusion Hb levels may create the opposite transfusion burden. In MDS, more liberal pretransfusion Hb levels should be targeted in patients with comorbidities. Studies should be conducted to demonstrate the reasons for not achieving target values, and remedial policies should be developed urgently. Mortality, QoL, thrombosis, and cost-effectiveness are open issues in the literature, and multicenter studies should be designed.

## Figures and Tables

**Table 1:** Patient characteristics.

Diagnosis	Female/male	Age, median (min–max)
Thalassemia (*n* = 83)		
- Thalassemia major (*n* = 67)	33/34	23 (18–53)
- Thalassemia intermedia (*n* = 16)	11/5	33 (22–83)
Myelodysplastic syndrome (*n* = 9)	4/5	73 (62–83)

**Table 2:** Variables affecting median annual transfusion in units and session in thalassemia major.

	Transfusion units in median	*p*	Transfusion sessions in median	*p*
Splenectomy (no/yes)	29 vs 22.5	**0.007**	16 vs 13	**0.014**
Age	*r* = −0.298	**0.014**	*r* = −0.234	0.056

*Note:* Bold values are significant.

**Table 3:** Impact of the pretransfusion Hb threshold on annual erythrocyte concentrate consumption in thalassemia major.

Pretransfusion Hb threshold (median, g/dL)	Annual EC units median (min-max)	*p*	Annual EC transfusion sessions median (min-max)	*p*
≥ 9	15 (8–24)	**0.009**	11 (4–14)	**0.020**
7–8,99	27 (31–48)		14 (8–24)	
< 7	19 (10–28)		9.5 (5–14)	

*Note:* Bold values are significant.

Abbreviation: EC, erythrocyte concentrate.

**Table 4:** Impact of the pretransfusion Hb threshold on annual erythrocyte concentrate consumption in thalassemia intermedia.

Pretransfusion Hb threshold (median, g/dL)	Annual EC units median (min-max)	*p*	Annual EC transfusion sessions median (min-max)	*p*
≥ 9	7 (4–7)	0.644	5 (3–5)	0.632
7–8,99	7 (4–13)		4 (2–8)	
< 7	6 (16–16)		3 (3–13)	

Abbreviation: EC, erythrocyte concentrate.

**Table 5:** Variables affecting median annual transfusion in units and session in MDS.

	Transfusion units in median	*p*	Transfusion sessions in median	*p*
Comorbidity (No/Yes)	20 vs 5.5	**0.032**	9 vs 3.5	0.063
Disease risk (low/high)	6 vs 16.5	0.262	3 vs. 8.5	**0.048**

*Note:* Bold values are significant.

## Data Availability

The data that support the findings of this study are available from the corresponding author upon reasonable request.

## References

[1] Lal A. (2020). Challenges in Chronic Transfusion for Patients with Thalassemia. *Hematology*.

[2] El-Beshlawy A., Dewedar H., Hindawi S. (2024). Management of Transfusion-dependent *β*-thalassemia (TDT): Expert Insights and Practical Overview from the Middle East. *Blood Reviews*.

[3] Hokland P., Daar S., Khair W. (2023). Thalassaemia-A Global View. *British Journal of Haematology*.

[4] Khandros E., Kwiatkowski J. L. (2019). Beta Thalassemia: Monitoring and New Treatment Approaches. *Hematology-Oncology Clinics of North America*.

[5] Brunner A. M., Leitch H. A., van de Loosdrecht A. A., Bonadies N. (2022). Management of Patients with Lower-Risk Myelodysplastic Syndromes. *Blood Cancer Journal*.

[6] Wood E. M., McQuilten Z. K. (2020). Outpatient Transfusions for Myelodysplastic Syndromes. *Hematology*.

[7] Lucero J., Al-Harbi S., Yee K. W. L. (2023). Management of Patients with Lower-Risk Myelodysplastic Neoplasms (MDS). *Current Oncology*.

[8] Farmakis D., Porter J., Taher A., Domenica Cappellini M., Angastiniotis M., Eleftheriou A. (2022). 2021 Thalassaemia International Federation Guidelines for the Management of Transfusion-dependent Thalassemia. *HemaSphere*.

[9] Lal A., Thalassemia B. D. (2020). Common Clinical Queries in Management. *Indian Journal of Pediatrics*.

[10] Ali S., Mumtaz S., Shakir H. A. (2021). Current Status of Beta-Thalassemia and its Treatment Strategies. *Molecular genetics & genomic medicine*.

[11] (2023). Hasta Kan Yönetimi Rehberi Modul 3-Dahili Hastaliklar.Pdf. https://dosyamerkez.saglik.gov.tr/Eklenti/44848/0/3-hasta-kan-yonetimi-rehberi-modul-3---dahili-hastaliklarpdf.pdf.

[12] Musallam K. M., Barella S., Origa R. (2024). Pretransfusion Hemoglobin Level and Mortality in Adults with Transfusion-dependent *β*-thalassemia. *Blood*.

[13] Scheinberg P. (2020). In Search for a Haemoglobin Threshold in Myelodysplastic Syndromes. *British Journal of Haematology*.

[14] Killick S. B., Ingram W., Culligan D. (2021). British Society for Haematology Guidelines for the Management of Adult Myelodysplastic Syndromes. *British Journal of Haematology*.

[15] Greenberg P. L., Tuechler H., Schanz J. (2012). Revised International Prognostic Scoring System for Myelodysplastic Syndromes. *Blood*.

[16] Saliba A. N., Musallam K. M., Taher A. T. (2023). How I Treat Non-transfusion-dependent *β*-thalassemia. *Blood*.

[17] Shah F. T., Sayani F., Trompeter S., Drasar E., Piga A. (2019). Challenges of Blood Transfusions in *β*-thalassemia. *Blood Reviews*.

[18] Viprakasit V., Gattermann N., Lee J. W. (2013). Geographical Variations in Current Clinical Practice on Transfusions and Iron Chelation Therapy across Various Transfusion-dependent Anaemias. *Blood transfusion*.

[19] Wanchaitanawong W., Tantiworawit A., Piriyakhuntorn P. (2021). The Association between Pre-transfusion Hemoglobin Levels and Thalassemia Complications. *Hematology*.

[20] Kim H. J., Hwang S. H., Oh H. B., Ko D. H. (2023). Transfusion Thresholds: the Need for a Patient-Centered Approach in Hematologic Disorders that Require Chronic Transfusion Therapy. *Blood research*.

[21] Stanworth S. J., Killick S., McQuilten Z. K. (2020). Red Cell Transfusion in Outpatients with Myelodysplastic Syndromes: a Feasibility and Exploratory Randomised Trial. *British Journal of Haematology*.

[22] Buckstein R., Callum J., Prica A. (2024). Red Cell Transfusion Thresholds in Outpatients with Myelodysplastic Syndromes: Results of a Pilot Randomized Trial RBC-ENHANCE. *Transfusion*.

[23] Vijenthira A., Starkman R., Lin Y. (2022). Multi-national Survey of Transfusion Experiences and Preferences of Patients with Myelodysplastic Syndrome. *Transfusion*.

[24] Guarente J., Tormey C. (2023). Transfusion Support of Patients with Myelodysplastic Syndromes. *Clinics in Laboratory Medicine*.

[25] Shallis R. M., Zeidan A. M. (2021). Management of the Older Patient with Myelodysplastic Syndrome. *Drugs & Aging*.

